# A quantitative meta-analysis and review of motor learning in the human brain

**DOI:** 10.1016/j.neuroimage.2012.11.020

**Published:** 2013-02-15

**Authors:** Robert M. Hardwick, Claudia Rottschy, R. Chris Miall, Simon B. Eickhoff

**Affiliations:** a Behavioural Brain Sciences, School of Psychology, University of Birmingham, UK; b Institute of Neuroscience and Medicine (INM-1), Research Center Jülich, Germany; c Department of Psychiatry, Psychotherapy and Psychosomatics, RWTH Aachen University, Aachen, Germany; d Institute of Clinical Neuroscience and Medical Psychology, Heinrich-Heine University, Düsseldorf, Germany; e Research Imaging Institute, University of Texas Health Science Center, San Antonio, TX, USA

**Keywords:** Activation likelihood estimation, Dorsal premotor cortex, Serial response time task, Sensorimotor learning, Sequence learning

## Abstract

Neuroimaging studies have improved our understanding of which brain structures are involved in motor learning. Despite this, questions remain regarding the areas that contribute consistently across paradigms with different task demands. For instance, sensorimotor tasks focus on learning novel movement kinematics and dynamics, while serial response time task (SRTT) variants focus on sequence learning. These differing task demands are likely to elicit quantifiably different patterns of neural activity on top of a potentially consistent core network. The current study identified consistent activations across 70 motor learning experiments using activation likelihood estimation (ALE) meta-analysis. A global analysis of all tasks revealed a bilateral cortical–subcortical network consistently underlying motor learning across tasks. Converging activations were revealed in the dorsal premotor cortex, supplementary motor cortex, primary motor cortex, primary somatosensory cortex, superior parietal lobule, thalamus, putamen and cerebellum. These activations were broadly consistent across task specific analyses that separated sensorimotor tasks and SRTT variants. Contrast analysis indicated that activity in the basal ganglia and cerebellum was significantly stronger for sensorimotor tasks, while activity in cortical structures and the thalamus was significantly stronger for SRTT variants. Additional conjunction analyses then indicated that the left dorsal premotor cortex was activated across all analyses considered, even when controlling for potential motor confounds. The highly consistent activation of the left dorsal premotor cortex suggests it is a critical node in the motor learning network.

## Highlights

► Activation likelihood estimation was used to analyze 70 motor learning experiments. ► Analysis revealed a cortico-subcortical network for motor learning. ► Consistent activations were found across subgroups with differing task demands. ► Left dorsal premotor cortex was identified as a key structure in motor learning.

## Introduction

Functional neuroimaging studies have been instrumental in determining the neuronal networks that underlie different types of motor learning tasks reviewed in Doyon et al. (2003). There is, however, little consensus regarding which areas of the brain are consistently activated during the acquisition of motor skills. This may be a result of the diverse range of experimental paradigms that have been used to examine motor learning. For instance, neuroimaging studies have examined a variety of sensorimotor paradigms, including learning dexterous skills such as playing musical instruments (Buccino et al., 2004) or tying knots (Tracy et al., 2003), learning visuomotor paradigms such as adapting movements in response to perturbations (Inoue et al., 2000; Nezafat et al., 2001), and phase coordination paradigms where participants learn to perform novel bimanual movement patterns (Puttemans et al., 2005; Rémy et al., 2008). Performance improvements in these sensorimotor tasks occur as participants learn to perform novel kinematics (movement speed and limb geometry) and/or dynamics (muscle forces and joint coordination). In contrast to sensorimotor tasks, variants of the serial reaction time task (SRTT; Nissen and Bullemer, 1987) are notable as they rely on responding to visual stimuli only by pressing a corresponding button. SRTT variants therefore represent *learning of sequential motor behavior*, yet have relatively *minimal demands on motor execution*, as participants respond through primarily isometric contractions of the finger muscles. Thus, while sensorimotor tasks and SRTT variants are both useful paradigms with which to examine the neural substrates that underlie motor learning, their actual task demands differ considerably; sensorimotor tasks have greater motor demands and emphasize the learning of novel movement kinematics and dynamics, while SRTT variants have relatively minimal motor demands and focus on learning sequential motor behavior. Identifying areas of diverging activation (i.e. those that are activated primarily by sensorimotor tasks or SRTT variants) will thus reveal activations relating to the specific demands of each task type. Conversely, areas that are consistently activated across both sensorimotor tasks and SRTT variants are likely to represent the ‘core’ network of brain structures that are essential for motor learning. Areas identified as being critical to motor learning could then be targeted using neurostimulation techniques, which have been used to modulate the function of brain structures in order to augment skill acquisition (Galea et al., 2011; Reis et al., 2009).

Previous qualitative reviews have highlighted both similarities and differences between the areas that underlie motor learning for sensorimotor tasks and SRTT variants. Robertson (2007) suggested both paradigms activate the striatum and cerebellum, but that sensorimotor paradigms are more dependent on cortical motor areas while SRTT variants place greater reliance on prefrontal areas. Hikosaka et al. (2002) describe a framework where two cortico-striatal-cerebellar loops underlie motor sequence learning. Performance improves rapidly during initial phases of learning, with changes being driven by an ‘associative’ loop. This loop comprises frontal, parietal and premotor cortical regions, the caudate, and associative cerebellar regions. The gradual increments in performance that occur later, however, are predominantly driven by a ‘motor’ loop. This loop consists of motor areas of the cortex, putamen, and cerebellum. The authors note, however, that this model may not hold for sensorimotor adaptation tasks such as learning to move against curl fields or learning visuomotor rotations. In contrast, Doyon and Benali (2005), Doyon et al. (2003, 2009) propose a single model that can encompass both types of task. Sensorimotor tasks such as motor adaptation primarily recruit a network of cortico-cerebellar structures, while sequence learning tasks instead involve greater contributions from a cortico-striatal system.

Despite the conflicting nature of these models, there is a degree of consensus regarding the specific roles that individual brain areas contribute to motor learning. The cerebellum is widely considered to maintain a ‘forward model’ of the motor apparatus, used to predict the sensory consequences of actions and detect errors in these predictions (Hikosaka et al., 2002; Krakauer and Mazzoni, 2011; Penhune and Steele, 2012; Shadmehr and Krakauer, 2008). Despite some controversy, it is widely suggested that the basal ganglia are implicated in probabilistic calculations and reward for optimal action selection (Hikosaka et al., 2002; Krakauer and Mazzoni, 2011; Penhune and Steele, 2012; Shadmehr and Krakauer, 2008). The primary motor cortex (M1) is consistently implicated in the use dependent acquisition and storage of muscle synergies required for faster and more precise movements (Krakauer and Mazzoni, 2011; Penhune and Steele, 2012; Shmuelof and Krakauer, 2011). The relatively consistent interpretations of the roles of the cerebellum, basal ganglia and motor cortex may stem from their highly preserved architecture in vertebrate species, which afford multiple converging sources of evidence as to their function (Shmuelof and Krakauer, 2011).

The roles of the parietal cortex and medial temporal lobe (MTL), however, are a matter of conjecture. Shadmehr and Krakauer (2008) suggest the parietal lobe combines the expected sensory consequences of movements (produced by the cerebellum; Miall and King, 2008; Miall et al., 2007) with actual sensory feedback to generate state estimations (see also Desmurget et al., 1999). Similarly, while Shadmehr and Krakauer (2008) indicate that the MTL is independent to the acquisition of motor skills, Robertson proposes that MTL engagement increases with temporal task demands, and that it is involved in learning ‘higher order’ components of sequences (see Robertson, 2007). It is, however, notable that Shadmehr and Krakauer (2008) primarily considered sensorimotor tasks, while Robertson (2007) was specifically reviewing SRTT variants. Task specific differences both between these experimental paradigms and within their specific demands may therefore explain the presence or absence of activity in the MTL and SPL.

Thus, previous reviews highlight consistent roles for the basal ganglia, cerebellum and M1, while bringing forth conflicting views on the roles of the parietal cortex and MTL. It should be considered that these reviews, though informative, are primarily qualitative in nature, usually drawing inference from the results of relatively few key studies. This leaves scope for quantitative techniques that examine evidence from a broader spectrum of studies to be utilized to assess which areas of the brain consistently contribute to motor learning.

Coordinate based meta-analyses can integrate the results of multiple neuroimaging studies across a field of research in a quantitative, unbiased fashion. Pooling data from multiple investigations provides opportunities to address several of the problems inherent to individual neuroimaging studies, such as their typically limited sample sizes (10–20 participants). Combining results from multiple investigations thus provides an opportunity to combat their relatively low statistical power. Individual neuroimaging studies are also sensitive to specific aspects of paradigm implementation and the particular contrasts examined. This can lead to diverging patterns of results between studies due to subtle differences in experimental design. Summarizing their results in a quantitative manner provides results that are less influenced by such study-specific ‘noise’. Activation Likelihood Estimation (ALE) provides a well established technique for quantitative voxelwise random effects meta-analysis (Eickhoff et al., 2009, 2012; Laird et al., 2005; Turkeltaub et al., 2002, 2012). This approach determines areas of significant spatial convergence based on peak activation coordinates reported in previous neuroimaging investigations. A key advantage of this coordinate-based approach is its principled statistical testing procedure against a null distribution to provide quantitative results.

The current investigation therefore utilized ALE to summarize the existing functional neuroimaging literature on motor learning and identify brain areas consistently activated during motor learning tasks. We hypothesized that an integration across all experiments would primarily reveal activations in motor cortical and cerebellar brain structures. Controlling for motor execution and for hand use during the tasks would allow further specification of areas that are involved in the higher level aspects of motor learning (as opposed to those simply involved in motor control). Furthermore, we hypothesized that while both sensorimotor and SRTT variant tasks would elicit activity in brain areas relating to motor preparation and execution, sensorimotor tasks would elicit greater activations in areas utilized to sense or predict the current state of the body and control movements. Finally, we hypothesized that areas that were demonstrated to be consistently activated across both sensorimotor and SRTT variant tasks would represent the ‘core’ areas essential to the motor learning network.

## Material and methods

### Data used for the meta-analysis

Studies to be integrated in the current meta-analysis were obtained via PubMed literature searches (www.pubmed.org, search strings “motor learning” or “sequence learning”) on functional magnetic resonance imaging (fMRI) and positron emission tomography (PET) experiments. Citations within these papers and previous qualitative reviews were examined to identify additional functional imaging studies to include in the analysis. Only publications reporting whole-brain analyses in standard reference space (Talairach/Tournoux, MNI) were included (coordinates reported in Talairach space were transformed into MNI coordinates using the Lancaster transformation; Laird et al., 2010; Lancaster et al., 2007). Only results from group analyses of healthy adult participants were considered for further analysis; single subject reports and between-group comparisons were excluded. In cases where studies reported data from patient populations, only data from healthy controls was used. The sample was further restricted to examine only manual motor learning tasks, as the number of studies examining training with other effectors (e.g. the legs) was insufficient for meaningful analysis. As an examination of the eligible studies revealed that relatively few experiments presented data on training related decreases in activity associated with motor learning, the meta-analyses presented here examined only training related increases in activation. We do, however, present a summary of the data from analyses of training related decreases in activity in the supplementary materials, as it has been proposed that they may reflect important changes in the recruitment of brain areas that underlie motor learning (see Dayan and Cohen, 2011).

Each analysis was accompanied by two subanalyses that controlled for potentially confounding factors. In each case, the first subanalysis examined only experiments performed with the right hand. This controlled for activations relating to a potential mixture of lateralization effects. The right hand was chosen as the majority of studies examined tasks using the right hand, allowing maintenance of adequate sample sizes. The second of each of the subanalyses controlled for movement execution. This allowed activations relating to motor performance to be separated from those more specifically related to motor learning. This was achieved by excluding contrasts that compared motor learning with a rest condition, leaving only contrasts that involved a movement matched control condition.

### Global analysis

70 experiments met the inclusion criteria. These experiments formed the basis for a global analysis; which was conducted as an integration of the entire current fMRI literature on motor-learning producing an objective, data driven overview. The global analysis included data from a total of 974 subjects and 954 activation foci. Details of the included experiments are presented in Supplementary Table 1.

The reporting of subject ages was not standardized across studies—some reported group age ranges, some reported mean group ages, and some reported both. We ascertained extreme values of 18–71 years from studies reporting age ranges. From the 39 studies that provided mean ages, we calculated a mean ± SD of 27.5 ± 10.5 years. This number should be viewed with caution, however, for several reasons. First, several studies did not report mean age of their participants. Second, there was usually a broad range of age-distribution within each study, at least judging from those that did report measures of dispersion. Finally, a few studies also focused on somewhat older participants, usually in their fifties (e.g., Daselaar et al., 2003; Naismith et al., 2010; Nakamura et al., 2001; Werheid et al., 2003). Consequently, we would argue that the observed convergence should represent motor-learning related effects in generally younger to middle-aged subjects, while specific claims related to a particular age-group or even on age-related effects are not warranted from this data.

A subanalysis examining only tasks performed with the right hand considered 39 experiments (456 subjects, 558 foci). A further subanalysis examining only contrasts that controlled for movement execution considered 47 experiments (695 subjects, 553 foci).

### Paradigm specific analyses

To examine differences between paradigm types, the entire pool of experiments was then separated into two subgroups: 35 ‘sensorimotor tasks’ and 35 ‘SRTT variants’. This distinction was based on the relative motor demands of the task. Sensorimotor tasks involved learning to produce new patterns of movement kinematics and/or dynamics. In contrast, SRTT variant tasks involved learning to perform skilled sequences of button presses with minimal novel motor components. All SRTT variant studies involved simple finger press responses to visual stimuli. There were, however, four key differences in implementation and analysis between SRTT variants. Firstly, the length of the repeating sequence varied between studies. Sequences consisted of between 5 and 18 items (Mean ± SD = 8.0 ± 3.3 items). Secondly, participants were not always made aware of the presence of a repeating sequence. In explicit SRTT variants participants were informed that a repeating sequence of stimuli and hence reactions would be present. In implicit SRTT variants participants were not informed that a repeating sequence would be present. Thirdly, the hand used to perform the task was also varied. Across experiments, participants performed SRTT tasks with the left hand, the right hand, or bimanually. Finally, the contrast examined differed between studies. Some studies compared the repeating sequence condition to a random sequence condition (thus controlling for activations related to movement execution). Other studies compared the performance in the repeating sequence condition to a rest condition, or to an un-modeled baseline. It should therefore be noted while all contrasts examined included a sequence-learning component, some also included components that could be related to ‘global’ aspects of learning (i.e. the improvements due to repeated performance and/or greater familiarity with the task). This factor was accounted for in a sub-analysis that controlled for movement execution (i.e. included only tasks comparing learning a repeating sequence to a condition with a random sequence, thus controlling for global learning). Supplementary Table 1 presents a summary of the details for each study.

#### Sensorimotor tasks

The analysis of 35 sensorimotor tasks considered 473 subjects and 323 foci. Further subanalyses examined 20 sensorimotor tasks performed with the right hand (243 subjects, 165 foci) and 23 sensorimotor tasks that controlled for movement execution (348 subjects, 217 foci).

#### SRTT variants

The analysis of 35 SRTT variants considered 501 subjects and 631 foci. Complementary subanalyses considered 19 SRTT variants performed with the right hand (213 subjects, 393 foci) and 24 SRTT variants that controlled for movement execution (347 subjects, 336 foci).

#### Contrast analyses: sensorimotor tasks vs SRTT variants

An additional contrast analysis was also performed on the data from the two subgroups of tasks. This analysis was used to determine whether specific clusters of activity were more frequently associated with either sensorimotor tasks or SRTT variants. A further contrast analysis compared activations during explicit and implicit SRTT variants. This analysis aimed to determine whether explicit awareness of the presence of a repeating sequence led to stronger activation during SRTT variant tasks.

#### Conjunction analysis

Using masks from the global analysis in order to achieve greater specificity, the results from the analyses of sensorimotor tasks and SRTT variants were then submitted to conjunction analysis. This allowed determination of which structures were consistently activated across both types of task. Conjunctions of subanalyses were also created to provide further controls against potential confounds.

#### Combined conjunction analysis: the ‘motor learning core’

In a final step, a combined conjunction analysis was conducted to determine which areas were activated regardless of task type and potential motor confounds. Areas surviving this conjunction are therefore likely to represent core areas for motor learning.

### Analysis procedure

Coordinate based meta-analyses were conducted using the revised version of the activation likelihood estimation (ALE) algorithm (Eickhoff et al., 2009; Turkeltaub et al., 2002). The ALE algorithm identifies converging areas of activity across different experiments, empirically determining whether this clustering is greater than expected by chance. ALE captures the spatial uncertainty associated with reported coordinates, treating them as the centers for 3D Gaussian probability distributions (Turkeltaub et al., 2002) with widths based on empirical between-subject and between-template comparisons (Eickhoff et al., 2009). The revised algorithm accounts for the increased spatial reliability of studies with larger sample sizes by modeling their activations using smaller Gaussian distributions (Eickhoff et al., 2009). The algorithm also accounts for comparisons between groups of unequal sizes by computing a null distribution under the assumption of label-exchangeability (Eickhoff et al., 2012).

All activation foci for a given experiment were combined for each voxel to produce a modeled activation map (MA map; Turkeltaub et al., 2012). ALE scores describing the convergence of coordinates for each location were then calculated via the union of individual MA maps. To distinguish areas where the convergence between studies was greater than would be expected by chance (i.e. to separate true convergence from noise) ALE scores were compared to a nonlinear histogram integration based on the frequency of distinct MA values (see Eickhoff et al., 2012). As functional activations occur predominantly in grey matter areas, ALE scores were computed only for voxels with a ≥ 10% probability of containing grey matter (Evans et al., 1994). Results were thresholded at p < 0.05 (cluster-level FWE, corrected for multiple comparisons, cluster-forming threshold at voxel level p < 0.001).

All contrast analyses were computed using the most recent version of the (random effects) ALE subtraction analysis (Eickhoff et al., 2012). Voxel-wise differences between ALE maps were first calculated for the two pools of experiments. The experiments contributing to either analysis were then pooled and randomly divided into two groups of equal size to the sets of contrasted experiments (Eickhoff and Grefkes, 2011; Eickhoff et al., 2012). Voxelwise ALE scores for these two randomly assembled groups were subtracted from each other and recorded, and this process was repeated 10,000 times to yield an empirical null distribution of ALE score differences between the two conditions. The map of differences based on this procedure was then thresholded at a posterior probability for true differences of P > 0.95 and inclusively masked by the respective main effect of the minuend (cf. Chase et al., 2011; Rottschy et al., 2012).

### Labeling

All results were anatomically labeled according to their most probable macroanatomical and cytoarchetectonic locations using the SPM Anatomy Toolbox (Eickhoff et al., 2005, 2006, 2007). Additional functional labels were obtained from a functional meta-analysis of cortical motor areas (Mayka et al., 2006). Peak maxima of the reported coordinates are presented in MNI space.

## Results

### Global analysis of all tasks

**Fig. 1:**
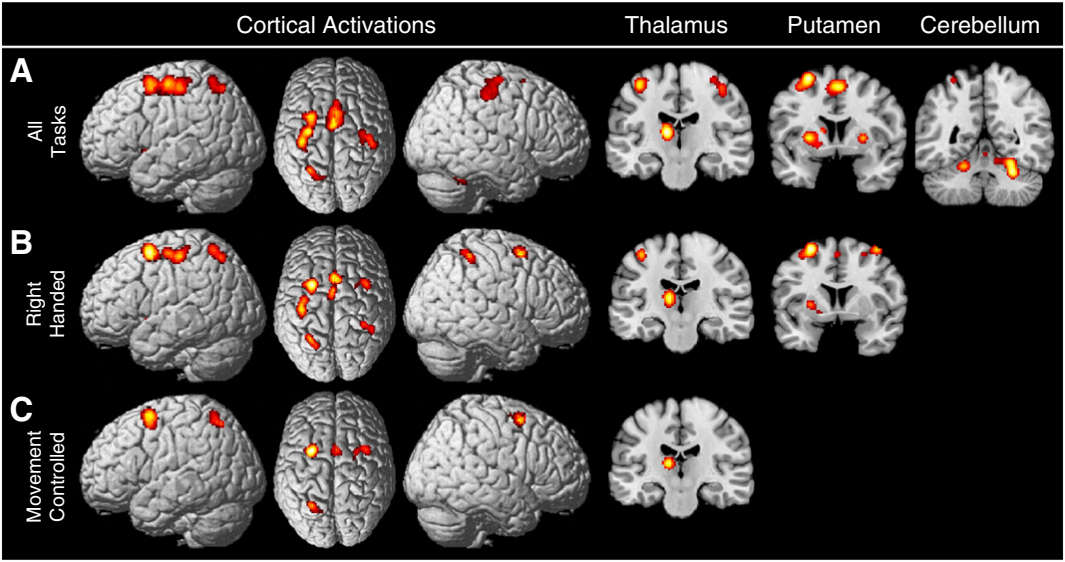
Results of the global analysis based on all motor learning experiments examined. (A) presents data from the analysis of all 70 experiments. Significant converging activity between studies was found in the left dPMC, pre-SMA, SMA proper, bilateral M1, left S1, left SPL, left thalamus, bilateral putamen, and bilateral/anterior medial cerebellum. Subanalyses controlled for potential confounds. (B) presents a subanalysis of 39 experiments that used the right hand. (C) presents a subanalysis results for 47 experiments that controlled for movement execution.

The global analysis examined all available studies in order to produce a data driven and objectively defined synthesis of the current neuroimaging literature on motor learning, and identify the network of brain areas that are commonly activated across all types of motor learning tasks. This global analysis examined all 70 experiments considered in the meta-analysis. Areas demonstrating significant convergence between experiments were the left dorsal premotor cortex (dPMC), bilateral supplementary motor cortex (SMC: comprising both the SMA-proper and pre-SMA), bilateral primary motor cortex (M1), left primary somatosensory cortex (S1), left superior parietal lobule (SPL), left thalamus, bilateral putamen, and multiple clusters in the cerebellum. These results are presented in Fig. 1A. Coordinates of local maxima for all activations as well as details on their histological allocation using probabilistic cytoarchitectonic maps are presented in Table 1.

Two subanalyses controlled for effects of hand use and movement execution. A subanalysis examining all 39 experiments in which participants performed tasks using only their right hand identified consistent activity in the bilateral dPMC and SMA as well as left M1, right S1, bilateral SPL, left thalamus, and left putamen (see Fig. 1B). A subanalysis that considered all 47 experiments that controlled for movement execution revealed activations in the bilateral dPMC, SMC, left SPL, and left thalamus (See Fig. 1C). Coordinates of local maxima for these activations are presented in Table 1.

### Paradigm specific analyses

Potential differences between paradigm subtypes were then considered, in particular, due to the differing task demands of sensorimotor tasks (which have greater motor demands and focus on learning novel movement kinematics and dynamics and kinematics) and SRTT variants (which have minimal novel motor demands and focus on learning sequential motor behavior).

#### Sensorimotor tasks

**Fig. 2:**
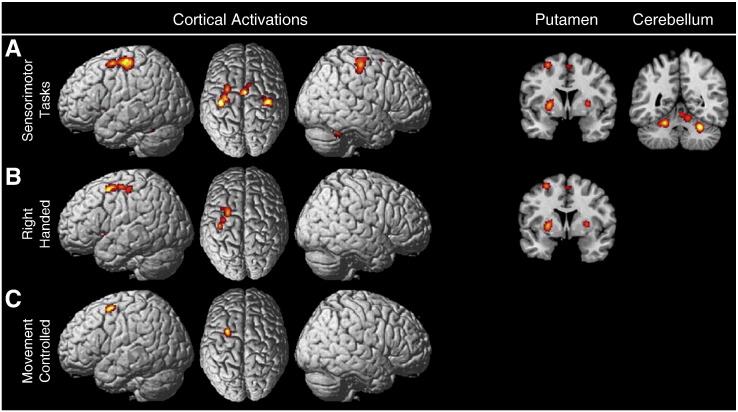
Results of the task specific analysis of sensorimotor tasks. (A) presents results from the 35 sensorimotor tasks examined. Significant convergence was found in the left dPMC, bilateral M1, SMA proper, bilateral putamen and bilateral/anterior medial cerebellum. Subanalyses controlled for potential confounds. (B) presents a subanalysis of the 20 sensorimotor experiments performed with the right hand. (C) presents a subanalysis of the 23 sensorimotor experiments that controlled for movement execution.

The analysis of 35 sensorimotor tasks revealed consistent activation in left dPMC and bilateral M1 as well as the bilateral putamen, and multiple areas of the cerebellum. These results are presented in Fig. 2A. A subanalysis of 20 right handed sensorimotor tasks revealed lateralized activity in the left dPMC, left M1 and bilateral putamen (see Fig. 2B). A further subanalysis of 23 sensorimotor tasks that controlled for movement execution revealed only activity in the left dPMC (see Fig. 2C). Peak activation foci for the sensorimotor analysis and subanalyses are presented in Table 2.

#### SRTT variants

**Fig. 3:**
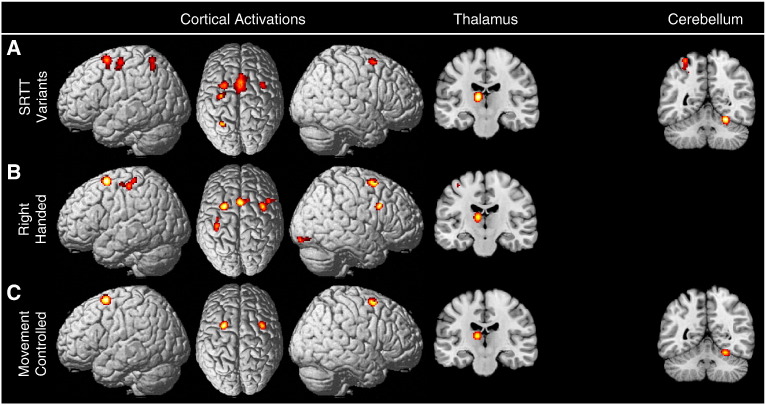
Results of the task specific analysis of SRTT variant tasks. (A) presents results from the analysis of all 35 SRTT variants examined. Significant convergence was revealed in the bilateral dPMC, left M1, pre-SMA, SMA proper, left SPL, left thalamus and right cerebellum. Subanalyses controlled for potential confounds. (B) Presents a subanalysis of the 19 SRTT variant experiments performed with the right hand. (C) Presents a subanalysis of the 24 SRTT variant experiments that controlled for movement execution.

Analysis of the 35 SRTT variants revealed significant convergence between cortical foci in bilateral dPMC and SMC as well as left M1, left SPL, left thalamus and right cerebellum (see Fig. 3A). A subanalysis restricted to the 19 SRTT variants performed using the right hand demonstrated significant activations in bilateral dPMC, right ventral PMC, bilateral SMC, left M1, left SPL, left thalamus and right cerebellum (see Fig. 3B). A second subanalysis considering only the 24 SRTT variant experiments that controlled for movement execution found activations in the bilateral dPMC, left thalamus, and right cerebellum (see Fig. 3C). Peak coordinates for the clusters identified in the SRTT variant analysis and subanalyses are presented in Table 3.

#### Contrast analysis: sensorimotor tasks vs SRTT variants

**Fig. 4:**
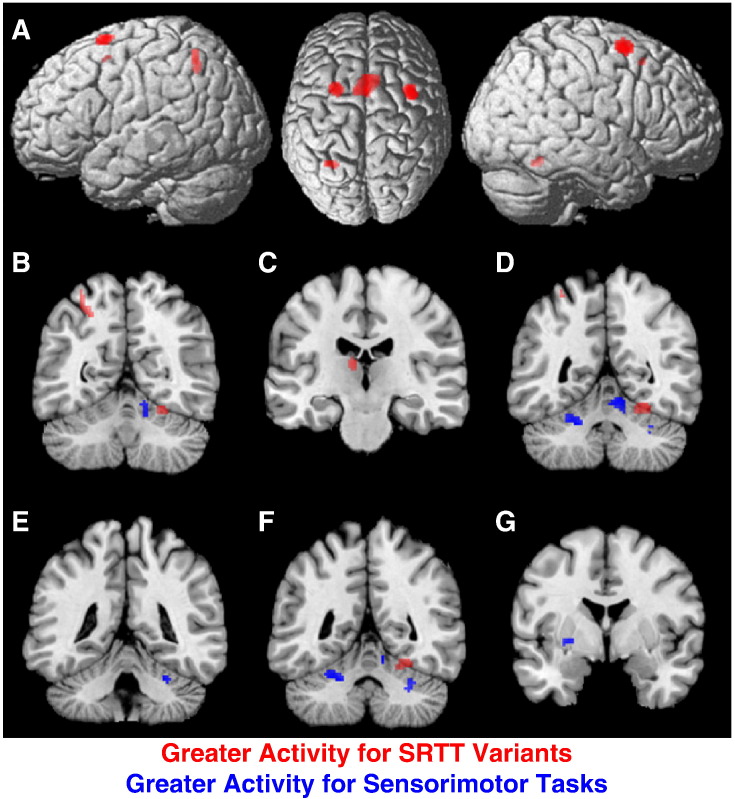
Results of the contrast analysis of secondary results comparing SRTT variants with sensorimotor tasks. Areas shown in red were more consistently involved in SRTT tasks, while areas shown in blue were more strongly implicated, i.e., more consistently activated, in sensorimotor tasks. SRTT variants elicited greater activity in cortical structures (A) with stronger activity in the dPMC, SMC, and SPL. SRTT tasks also elicited strong activity in a small cluster in the right lateral cerebellum (B) and left thalamus (C). Sensorimotor tasks elicited greater activity within the right cerebellar vermis (D), another small cluster in the right lateral cerebellum (E) and a cluster in the left lateral cerebellum (F). Sensorimotor tasks also elicited greater activity in the left putamen (G).

As the next step, a contrast analysis was performed between the two pools of results from the task specific analyses, i.e., sensorimotor tasks and SRTT variants. This contrast analysis aimed to determine where in the brain activation was more strongly associated with sensorimotor tasks or SRTT variants. Greater convergence for SRTT variants than for sensorimotor tasks was found in multiple cortical areas, namely the bilateral dPMC, SMA and SPL (see Fig. 4A). Subcortically, stronger convergence for the SRTT tasks was found within the left thalamus, and a small cluster in the right cerebellum (Figs. 4B–C). In contrast, multiple bilateral and superior medial areas of the cerebellum were found to demonstrate stronger convergence for sensorimotor as compared to SRTT tasks (Figs. 4D–F). Activation within the basal ganglia was also significantly more strongly associated with sensorimotor tasks (Fig. 4G).

#### Contrast analysis: implicit SRTT variants vs explicit SRTT variants

**Fig. 5:**
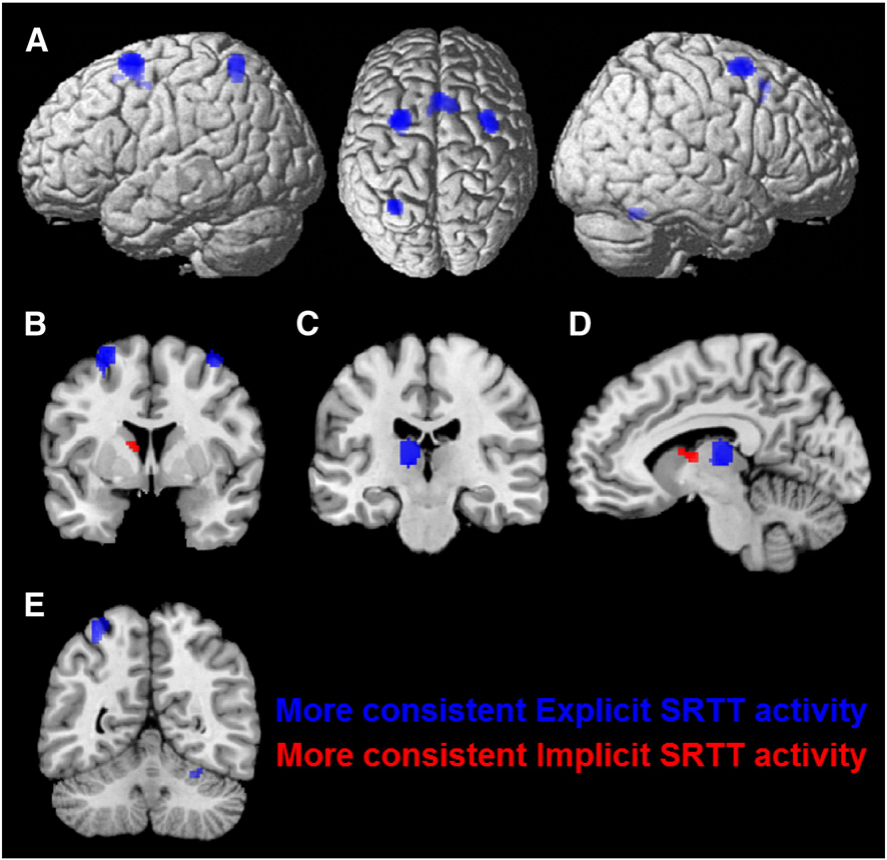
Results of a contrast analysis between 17 explicit and 15 implicit SRTT variants. Areas shown in blue were involved more consistently activated in explicit SRTT variants, while areas shown in red were implicated more strongly, i.e., activated more consistently, in implicit SRTT variants. A) More consistent cortical activation for explicit SRTT variants in the bilateral dPMC, SMC and SPL. B) Higher convergence for implicit SRTT variants in the rostral thalamus C) higher convergence for explicit SRTT variants in the caudal thalamus. D) Illustrates the relative positions of the activations shown in subpanels B and C. E) Presents more consistent activation for the explicit SRTT in the right lateral cerebellum. The stronger overall pattern of activity for explicit SRTT variants may be due to the participant's explicit awareness of the sequence to be learned.

A further contrast analysis compared activations during explicit and implicit SRTT variants. This analysis revealed that activations in the bilateral dPMC and SMA, as well as in left SPL and thalamus were stronger when participants had explicit awareness of the presence of a repeating sequence, whereas only the head of the left caudate was more consistently recruited in implicit tasks (see Fig. 5).

#### Conjunction analyses

**Fig. 6:**
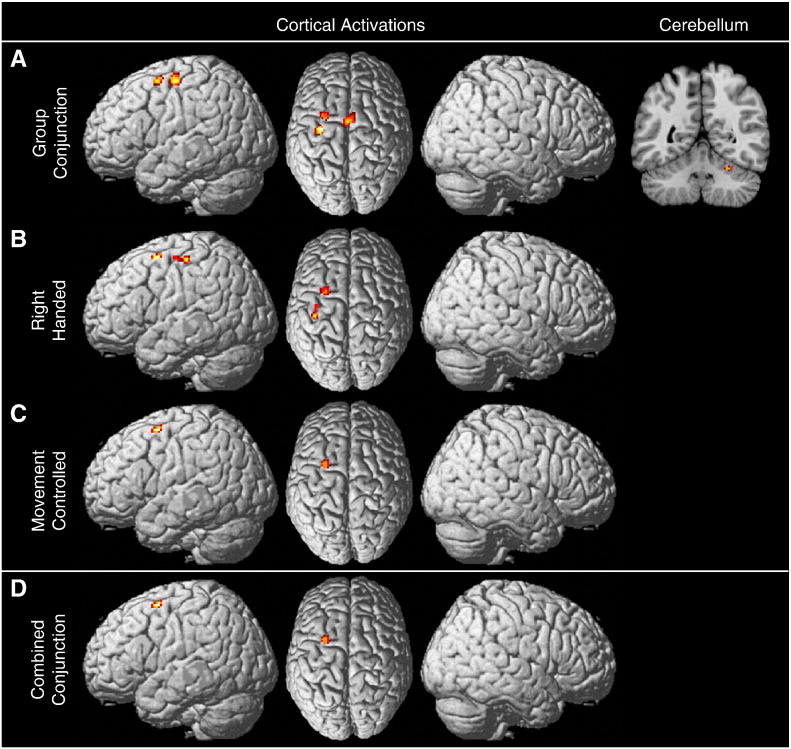
Results of conjunction analyses. (A) presents a conjunction of the global, sensorimotor and SRTT variant analyses (i.e. conjunction of Figs. 1A, 2A and 3A). Clusters in the SMA, left dPMC and left M1 survived the conjunction, and are thus consistently activated across both sub-groups of paradigms. (B) presents a conjunction of the right handed subanalyses (i.e. conjunction of Figs. 1B, 2B and 3B). (C) presents a conjunction of the movement controlled subanalyses (i.e. conjunction of Figs. 1C, 2C and 3C). (D) presents a combined conjunction analysis (i.e. conjunction of A, B and C above). Results of the combined conjunction analysis indicate that the left dPMC is consistently activated across different motor learning tasks regardless of potential motor confounds.

A next step was to determine which areas were consistently activated across both subgroups of paradigms. Using the global analysis as a mask to provide greater specificity, a conjunction analysis was conducted across the task specific analyses of sensorimotor tasks and SRTT variants (the conjunction was thus based upon the Figs. 1A, 2A and 3A). Clusters that survived this conjunction (and were hence consistently associated with motor learning across sensorimotor tasks and SRTT variants) were found in left dPMC, left M1, SMA proper and lobule VI of the right cerebellum (see Table 4). These activations are presented in Fig. 6A.

Conjunctions of data from the subanalyses were also conducted. Using the results of the global analysis of all right handed tasks as a mask, a conjunction was computed over the right handed subanalyses for sensorimotor tasks and SRTT variants (i.e. Figs. 1B, 2B and 3B). This conjunction analysis of right handed tasks revealed consistent converging activation in the left dPMC and left M1. The results of this conjunction are presented in Fig. 6B. Peak coordinates for this conjunction analysis are reported in Table 4. A conjunction of the movement controlled subanalyses was also conducted. This analysis, masked using the results of the movement controlled global analysis, was performed as a conjunction across the results from the motion-controlled analyses of sensorimotor tasks and SRTT variants (i.e. Figs. 1C, 2C and 3C). This revealed activation only in the left dPMC (see Fig. 6C, Table 4).

#### Combined conjunction analysis: the ‘motor learning core’

This final step aimed to identify brain areas that represented the core structures for motor learning. This analysis was generated by creating a conjunction of results from the previous conjunction analyses (i.e. conjunction Figs. 6A, B and C). The conjunction of their results revealed consistent activation of the left dPMC (peak coordinates in MNI space: − 26, 4, 60, activation presented in Fig. 6D).

To assess the reliability of this activation, we conducted a highly conservative supplementary analysis examining only tasks that both used the right hand and controlled for movement execution (12 experiments). Like the combined conjunction analysis described above, this revealed activation of left dPMC when performing a conjunction across SRTT and sensorimotor tasks (see Supplementary Fig. 1). The results of the combined conjunction analysis, coupled with the highly conservative supplementary analysis, demonstrate the robust and consistent nature of the cluster of activity found in the left dPMC.

## Discussion

The study presented here assessed the network of brain areas that were consistently activated across 70 motor learning experiments. A global analysis revealed converging activations in dPMC, SMC, M1, S1, SPL, thalamus, putamen and the cerebellum. Further paradigm specific analyses indicated that these patterns of activity were similar across both sensorimotor tasks and SRTT variants. A contrast analysis, however, indicated that sensorimotor tasks led to more consistent activations in the left basal ganglia and bilateral cerebellum, while SRTT variants elicited more consistent activations in the bilateral dPMC, SMC, left SPL and left thalamus. A conjunction analysis nevertheless revealed a shared sub-network composed of the left dPMC, left M1, SMC and lobule VI of the right cerebellum was consistently recruited by both subgroups of task. Thus, while the contrast analysis indicated differences in activation likelihood, the overall pattern of results from the global and paradigm specific analyses demonstrate that a broadly similar network of structures underlies motor learning and performance across sensorimotor tasks and SRTT variants. Here we review the current evidence for the most likely roles of each of the structures identified in the meta-analysis. We also compare how the pattern of activation identified by the meta-analysis fits with previous models of motor learning.

### Dorsal premotor cortex

While the meta-analysis revealed consistent converging activity in the dPMC, the laterality of its activation was highly task dependent. Sensorimotor tasks revealed convergence only in the left dPMC, while SRTT variants elicited converging bilateral dPMC activity. Subanalyses considering only experiments performed with the right hand revealed the same pattern of left unilateral activation for sensorimotor tasks and bilateral dPMC activation for SRTT variants. This indicates that the pattern of activation revealed was not simply a result of differences in hand use—instead, the diverging patterns of activity are likely due to the differing demands of the sensorimotor task and SRTT variant subgroups. Schubotz and von Cramon (2003) have previously noted that hemispheric differences in PMC activity for SRTT variants may be accounted for by different aspects of learning. They suggest that the left PMC is activated during sequence acquisition, while the right PMC is involved during advanced stages of learning and in storage of sequences. Furthermore, the right PMC has been found to be active when participants learn purely perceptual components of the SRTT (Schubotz and von Cramon, 2002a,b). This is of particular relevance as the right dPMC was activated in subanalyses that controlled for movement execution. The consistent activation of the right dPMC during SRTT variants could therefore be attributed to participants learning advanced or perceptual (rather than movement execution related) aspects of sequence learning tasks.

The cluster of activity in the left dPMC was highly consistent, surviving the conjunction of analyses and the highly conservative supplementary analysis. The consistency of this activation indicates that the left dPMC is a structure of key importance for motor learning. In primates, the dPMC has both reciprocal connections with M1 and direct descending spinal projections, yet has a limited ability to directly contribute to movement execution (Boudrias et al., 2010; Dum and Strick, 2005). These data thus indicate that the dPMC contributes to motor learning at a level above movement performance. Previous literature also indicates that the dPMC may contribute to motor learning by selecting appropriate responses according to visual cues (Kalaska and Crammond, 1995; Picton et al., 2007). In particular, the left dPMC appears to play a dominant role in movement selection (cf. Bestmann et al., 2008); patients with lesions of the left dPMC show impaired response selection with either hand (Halsband et al., 1993; Rushworth et al., 1998), and TMS studies in healthy participants show that disrupting the left dPMC in healthy participants leads to increased reaction times when responding with either hand (O'Shea et al., 2007; Rushworth et al., 2003). TMS of the left dPMC has also been demonstrated to disrupt the online updating of movements (Lee and van Donkelaar, 2006). These multiple sources of converging evidence indicate that the dPMC plays a key role in the selection and updating of appropriate responses with either hand according to visual cues. These sources of evidence indicate that the left dPMC is an important structure for the visuomotor control of movement.

**Fig. 7:**
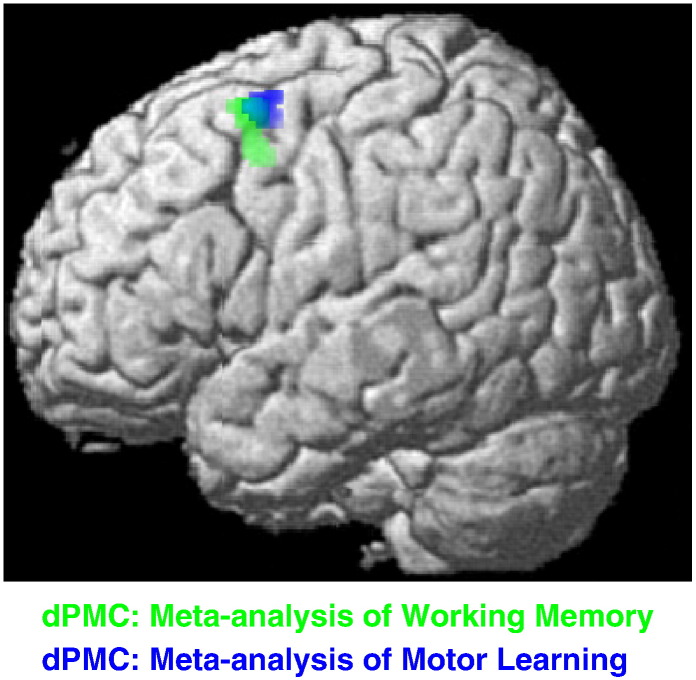
Further analysis of the results for the left dPMC, illustrating the clusters of left dPMC activation revealed in the combined conjunction analysis of motor learning tasks presented here (blue), and the area of the left dPMC found to play a role in spatial encoding in Rottschy et al. (2012) meta-analysis of working memory (green). The overlapping nature of the activations and their relative positions are consistent with a rostral/caudal gradation in dPMC function (see Fujii et al., 2000; Schubotz and von Cramon, 2003).

There is moreover evidence that the dPMC exhibits a continuum of visuomotor function. Rostral premotor areas contain a greater proportion of neurons with sensory properties while caudal premotor areas are more frequently associated with motor properties (see Fujii et al., 2000; Schubotz and von Cramon, 2003). This distinction is of particular relevance when considered in relation to a recent meta-analysis of working memory (see Rottschy et al., 2012). Their results indicate that the left dPMC was specifically involved in encoding visuospatial information during working memory tasks. The locations of the left dPMC activation found by Rottschy et al. (2012) and the left dPMC cluster found in the combined conjunction in the present meta-analysis are presented in Fig. 7. While the clusters overlap, it is notable that the working memory related cluster was in a more rostral location. In comparison, the activation from the present meta-analysis of motor learning was in a relatively caudal position. These patterns of activity are therefore consistent with the left dPMC exhibiting a rostrocaudal continuum of activity from sensory to motor functions (Fujii et al., 2000; Schubotz and von Cramon, 2003).

In summary, our results provide evidence to support a functional lateralization of the dPMC. The right dPMC was only reliably activated during SRTT variants, which may be attributed to the role the right dPMC plays in the learning and storage of perceptual sequences. In contrast, the left dPMC was consistently activated across all analyses examined. Subanalyses indicated this pattern of activation occurred independently of movement execution and hand use. Comparisons with a previous meta-analysis of working memory indicated a rostrocaudal continuum of left dPMC activity from sensory to motor functions. Accordingly, activity of the left dPMC may relate to a dominant role in movement selection via visuomotor integration. This important role in visuomotor processing indicates that the left dPMC is a key structure in the network of brain areas that underlie motor learning.

### Supplementary motor cortex

The main analysis of all motor learning tasks revealed activity in both the SMA proper and the pre-SMA. This activity is consistent with the role of the SMC and adjacent cingulated regions in the self initiation of voluntary movements (Deecke and Kornhuber, 1978; Hoffstaedter et al., 2012). Task specific analyses indicated sensorimotor tasks activated only the SMA-proper, while SRTT variants activated both the SMA-proper and pre-SMA. The primate pre-SMA contains cells that respond to specific sequences of movements (Clower and Alexander, 1998; Shima and Tanji, 2000; Tanji and Shima, 1994), appearing to be important for linking conditional rules to actions. This role in sequence learning may explain why the pre-SMA was only consistently engaged during SRTT variants (which focus on learning sequences). Rostrally positioned (pre-SMA) areas are frequently involved in non-motor cognitive processes (Leek and Johnston, 2009; Tanaka et al., 2005). Activation of the pre-SMA during SRTT variants could therefore be attributed to their (more cognitive) task demands when compared to sensorimotor paradigms. Conversely, a recent study indicates that stimulation of the SMA-proper and not the pre-SMA can enhance motor learning in a sensorimotor task with a repeating sequential component (Vollerman et al., in press). It should, however, be noted that while both this task and SRTT variants include a sequential component, the requirements of the pinch task could be considered to have a greater weighting towards motor-execution related than cognitive aspects of motor learning. More caudally positioned (SMA-proper) regions of the SMC are linked with motor functions such as volitional action execution (Jakobs et al., 2009; Nachev et al., 2008). This is consistent with the activation of the SMA-proper across both SRTT variants and sensorimotor tasks. It is also notable that the SMC and dPMC, which together comprise the classical Brodmann area 6, both demonstrate a rostrocaudal shift of SMC activity from cognitive to motor function.

### Primary motor cortex

Similarly to the dPMC, M1 activations were revealed in both the global and task specific analyses, but the laterality of the activation depended greatly upon the subgroup of tasks examined. Bilateral M1 activations were present in the analysis of sensorimotor tasks, while SRTT variants revealed only left lateralized M1 activity. A subanalysis examining only tasks performed with the right hand revealed activity in the left M1 alone for both the SRTT variant and sensorimotor task subgroups. This is consistent with M1 activity reflecting use of the contralateral hand. Finally, no converging activations were found in M1 when an analysis was conducted only on tasks that controlled for movement execution. These results demonstrate that M1's involvement in motor learning occurs predominantly at the level of movement execution. This evidence appears to indicate that M1 plays a generally subservient role in motor learning. In spite of this, it should be noted that M1 has also been argued to be involved in the retention of learned movements via their repeated performance (Classen et al., 1998; Galea and Celnik, 2009; Galea et al., 2011; Hadipour-Niktarash et al., 2007; Orban de Xivry et al., 2011; Reis et al., 2009; Zhang et al., 2011). In particular, several models of motor learning consider muscle synergies to be instrumental in reducing movement variability in later learning to allow improved levels of skilled performance (Krakauer and Mazzoni, 2011; Penhune and Steele, 2012; Shmuelof and Krakauer, 2011). Hence, M1 may play a role in motor learning through use dependent mechanisms above the pure executive function indicated by our analyses.

### Primary somatosensory cortex

The global analysis revealed consistent loci of activity in S1. Task specific analyses indicated that this was driven by activity from sensorimotor learning tasks. While this may simply reflect that greater levels of sensory feedback are present during tasks with greater movement based demands, there is also evidence to suggest an active role for S1 during motor learning. Accurate sensory feedback comprises an important component of the forward modeling process thought to underlie motor learning through the correction of sensory prediction errors (for a recent review see Hardwick et al., in press). In animal models, somatosensory lesions have been demonstrated to impair the acquisition of new motor skills (Pavildes et al., 1993). In humans, TMS studies have demonstrated that disrupting the normal function of S1 can interfere with motor learning when accurate sensory feedback is an important factor in performance (Vidoni et al., 2010), and can even enhance performance when proprioceptive and visual feedback are in conflict (Balslev et al., 2004). These data thus seem to indicate that S1 may contribute to motor learning by processing sensory feedback information relevant to mechanisms of learning through error detection and correction.

### Superior parietal lobule

Consistent activity of the left SPL (area 7A) was revealed in the global analysis of all tasks examined, and in the task specific analysis of SRTT variants. Subanalyses examining experiments performed with the right hand alone revealed similar patterns of activation: Bilateral area 7A activity was found when all right handed tasks were considered, but this activity was specific to the left hemisphere for SRTT variant tasks. In the subanalysis that controlled for movement execution, the left SPL was consistently activated in only the global analysis. The SPL is widely implicated in representation of the hands (Battaglia-Mayer et al., 2007; Connolly et al., 2003; Glover et al., 2005; Mountcastle et al., 1975; Rizzolatti et al., 1998). This focus on hand representation and the intimate interactions with the (dorsal) visual system may account for the consistent activity of the SPL in SRTT variants, which emphasize somatomotor hand control in response to visual stimuli. We found no consistent SPL activity in for sensorimotor tasks, which may reflect differences in parietal activation across the range of sensorimotor paradigms that have been examined. However, Hikosaka et al. (2002) have proposed that their model of a motor loop including the parietal cortex may not apply to certain sensorimotor tasks such as adaptation.

Only areas of the dPMC and SPL were found to be activated in subanalyses that controlled for movement execution. The primate dPMC receives afferent inputs from the SPL (Matelli et al., 1998), and these regions work in close cooperation to allow visuomotor control (Wise et al., 1997). Transforming sensory input to motor output thus appears to involve a route from the SPL to M1 via the dPMC (Johnson et al., 1993, 1996, cf. Cieslik et al., 2010, 2011). Data from the present meta-analysis therefore supports the suggestion that the SPL integrates visual and somatosensory inputs, routing multimodal sensorimotor outputs to the dPMC, which in turn represents the key hub for motor learning and provides preparatory motor input into M1.

### Thalamus and putamen

**Fig. 8:**
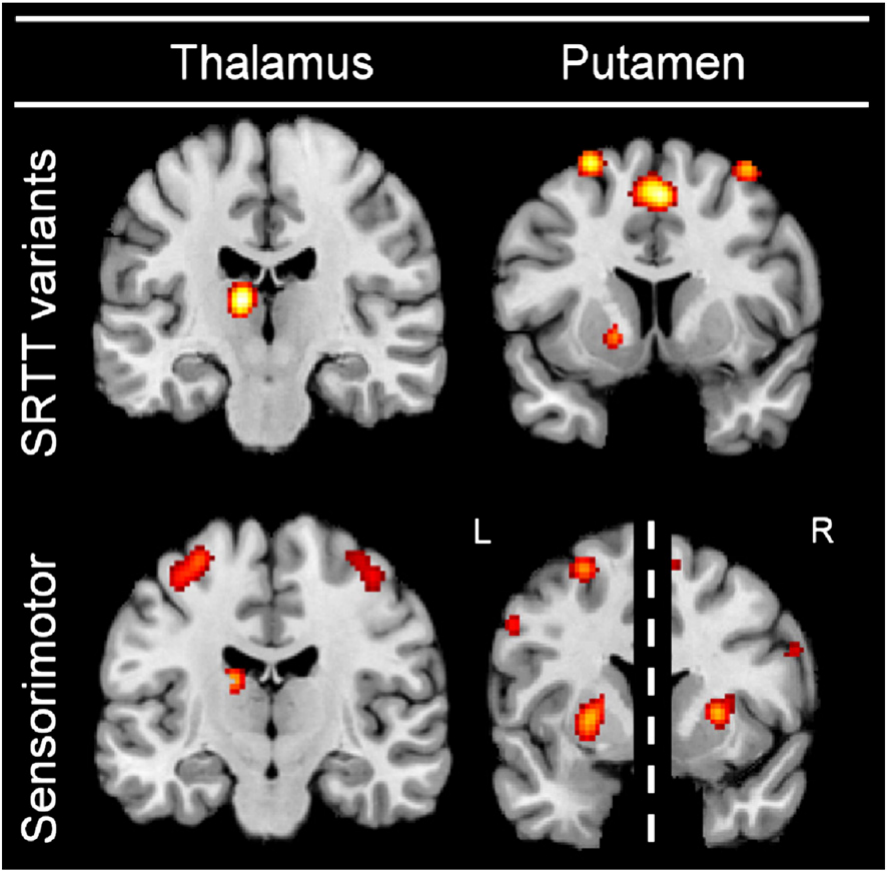
Further analysis of the results for the thalamus and putamen. Results presented at the cluster level uncorrected significance level indicate that both SRTT variants and sensorimotor tasks activated both of these subcortical areas. Dashed line between putamen results for sensorimotor tasks indicates differing x locations of peak maxima in MNI space.

While the global analysis revealed significant convergence in both the thalamus and putamen, task specific analyses suggested an apparent double dissociation. Analysis of sensorimotor tasks demonstrated significant converging activity in the putamen alone, while analysis of SRTT variants revealed significant converging activity only in the thalamus. Further investigation, however, indicated that both regions are activated by both task types. If a true double dissociation existed, it would be expected that the clusters identified in the global analysis of all tasks would be due to SRTT variants alone in the case of the thalamus, and related to sensorimotor tasks alone in the case of the putamen. Examination of these clusters revealed that this was not the case. In particular, the respective “preferred” type of task accounted for the majority of the activation probability (SRTT variants accounted for 71% of activity in the thalamus, while sensorimotor tasks accounted for 56/61% of activity in the left/right putamen). Thus both task types accounted for the majority of activation in the respective area, but also contributed approximately 30–45% of the activity of the other cluster. Further evidence indicating that both task types contributed to activity in both the thalamus and putamen was provided when data from the task specific analyses was considered at a reduced significance threshold (cluster-level uncorrected p < 0.05, while keeping the cluster-forming threshold at p < 0.001 at voxel level). Results indicated activity in both the Thalamus and the Putamen for both SRTT variants and sensorimotor tasks (see Fig. 8). Together, these sources of evidence indicate that both subgroups of tasks activate the thalamus as well as the putamen. Thus, while our results provide evidence for preferential activation, they do not represent a clear double-dissociation. Our results implicate a cortico-subcortical ‘motor circuit’ (Mazzoni and Bracewell, 2010; Postuma and Dagher, 2006), and are thus broadly consistent with models proposing that cortico-subcortical loops underlie motor learning (Doyon et al., 2003, 2009; Hikosaka et al., 2002).

### Cerebellum

The global analysis of all tasks revealed clusters of bilateral activity occurring in the lateral Cerebellum (lobules V–VI), and a further cluster in the right cerebellar vermis (lobules I–IV). Task specific analyses, however, demonstrated that only activity in the right lateral cerebellum was consistent across both sensorimotor and SRTT variants. In contrast, activity in the left lateral cerebellum and vermis were present only in sensorimotor tasks. The cerebellum was more active during sensorimotor tasks, consistent with its role in the “state estimation” process whereby predicted sensory consequences of actions are compared with actual sensory feedback (for a review see Hardwick et al., in press). State estimation allows the detection of prediction errors, which are essential for the adaptation and improved control of fast and accurate movements using feedforward control (Manto et al., 1994; Miall and King, 2008; Miall et al., 2007; Tseng et al., 2007). Previous research indicates the cerebellum plays a key role in the correction of prediction errors (Galea et al., 2011; Tseng et al., 2007), though only when large errors are introduced in a stepwise manner (Criscimagna-Hemminger et al., 2010). It is notable that the need for state estimation during sensorimotor tasks would be relatively high, as they involve learning novel patterns of movement. In contrast, the need for state estimation during SRTT variants would be relatively low due to the minimal motor component of the task (i.e. participants respond via primarily isometric button presses). Thus, we attribute the patterns of cerebellar activity revealed during sensorimotor tasks and SRTT variants to their differing demands on the process of state estimation.

### Comparison with previous models of motor learning

Several previously established models propose that cortico-subcortical loops underlie motor learning tasks. Doyon et al. (2003, 2009) propose that a network of cortico-cerebellar structures primarily underlies learning in sensorimotor tasks, while a cortico-striatal system primarily underlies sequence learning tasks. Results of the present meta-analysis are in general agreement with this proposal. While both sensorimotor tasks and SRTT variant task paradigms relied on similar networks, the performed contrast analysis indicated that the cerebellum was more strongly associated with sensorimotor tasks, while the thalamus was more strongly associated with SRTT variants. Putamen recruitment was, however, contradictory to this model, being more closely linked with sensorimotor tasks.

Hikosaka et al. (2002) propose a somewhat contrasting framework with dual cortico-striatal-cerebellar loops for the associative and motor aspects of learning. While Hikosaka et al. (2002) limit this proposal to the acquisition and refinement of sequences, the present meta-analysis is in broad agreement with a logical extension of this model. In this context, it should be considered that SRTT variants are more likely to rely on the associative loop (as they involve strengthening stimulus–response associations) while sensorimotor tasks are likely to rely more on the motor loop (as they involve refinement of motor performance). The similar patterns of cortical and cerebellar activity elicited during sensorimotor tasks and SRTT variants fit well with this premise. While the main analysis indicates the presence of a potential dichotomy in thalamus and putamen activity, further analyses indicated that both task types activate both structures (see “Thalamus and putamen” subsection of discussion and Fig. 8 for details).

Activations revealed in the present meta-analysis are therefore consistent with the proposal that cortico-cerebellar loops underlie motor learning (Doyon et al., 2003, 2009). The manner in which the identified areas work together is, however, still open to interpretation. Future investigations that model connectivity between the regions identified in the present meta-analysis will elucidate this issue.

Conflicting opinions exist regarding the involvement of the MTL in motor learning (Robertson, 2007; Shadmehr and Krakauer, 2008). The results of the present meta-analysis indicate that the MTL is not part of the core network of brain areas that underlie motor learning for either sensorimotor tasks or SRTT variants. This finding could be considered as evidence that motor learning occurs independently of the MTL (Shadmehr and Krakauer, 2008). For an alternative viewpoint, it is of interest to consider that Robertson (2007) proposes MTL engagement increases as the temporal and higher order demands of sequence learning tasks increase. It could, therefore be suggested that the series of SRTT variants included in the present meta-analysis did not have sufficient temporal or higher-order demands for the MTL to be consistently engaged across a majority of tasks.

### Methodological considerations

While the tasks included in the present meta-analysis were divided into two subgroups (sensorimotor tasks, SRTT variants) of roughly equivalent size, the sensorimotor tasks presented a less homogenous group than the SRTT variants. The former could be considered an amalgamation of a number of smaller groups of paradigms (i.e. dexterous tasks, visuomotor tasks and phase coordination tasks). Unfortunately, the relatively low number of studies within each of these smaller groups would not have provided enough data to produce reliable results.

Coordinate based meta-analyses of neuroimaging data pool results from studies that use similar contrasts. While a large majority of papers retrieved in the initial literature search presented data reporting activity related signal increases that occurred with motor training, only 1 in 3 studies reported training related decreases in activity. This represents an unfortunate imbalance in the literature, as examining training related decreases in activity in response to motor learning is of interest for several reasons. Firstly, motor training has been demonstrated to reduce the overall excitability threshold of the motor cortex (Pascual-Leone et al., 1995), indicating that training may lead to more efficient recruitment of motor structures. Secondly, it has been demonstrated that different areas of the brain are recruited during different stages of motor learning (Shadmehr and Holcomb, 1997). In this manner, decreasing activity revealed during fMRI studies may reflect changes due to increased efficiency, reduced errors, reduced co-contractions, and longer term changes underlying consolidation of learning. This underreporting of training related decreases in the literature may have occurred because experimenters simply did not consider investigating training related decreases. Alternatively, it could be due to underreporting of analyses that considered training related decreases which found null results, or results that were difficult to interpret.

A final consideration is that coordinate based meta-analyses are largely insensitive to the time course of activations, which may play an important role in motor learning. For instance, evidence from sensorimotor learning tasks suggest that the cerebellum is activated during initial phases of learning when error is high, but shows reduced activity during later phases of learning when error is low (see Nezafat et al., 2001). While coordinate based meta-analyses combine data from multiple studies to allow for robust inference on convergent findings, they are more likely to miss such temporal dynamics, as many studies do not report separate analyses related to time-dependent changes. Consequently, data will be pooled across different parts of the time-dependent changes during motor-learning. Thus, the results of the present study do not allow us to test the proposal that brain structures will contribute differentially to motor learning over time (see Danielle et al., 2011; Dayan and Cohen, 2011; Grafton et al., 1995, 2002; Hikosaka et al., 2002).

## Conclusions

The ALE meta-analysis presented here for the first time allowed the quantitative integration of the current neuroimaging literature on motor learning. It revealed consistent activity across paradigms in the dPMC, SMC, M1, S1, SPL, thalamus, putamen and cerebellum. This pattern of activations is broadly consistent with previously proposed models of the key structures involved in motor learning (Krakauer and Mazzoni, 2011; Penhune and Steele, 2012; Shadmehr and Krakauer, 2008; Shmuelof and Krakauer, 2011), and the proposal cortico-subcortical loops may drive motor learning (Doyon et al., 2003, 2009; Hikosaka et al., 2002).

Contrast analyses indicated that activity in the basal ganglia and cerebellum were more frequently (though not exclusively) associated with sensorimotor tasks, consistent with their proposed roles in selective reinforcement of motor programs and the detection of sensorimotor prediction errors. In comparison, activity of the dPMC, SMC, SPL and thalamus was more consistently associated with SRTT variants, consistent with their proposed roles in visuomotor integration and response selection. Despite these differences, a conjunction analysis of our global and task specific analyses revealed that the left dPMC, SMC, left M1 and right cerebellar areas were consistently activated across both SRTT variants and sensorimotor tasks. These results indicate that a consistent network of neural structures contributes to motor learning and performance across a range of paradigms despite their varying kinematic and dynamic demands. A result of particular interest was the finding that the left dPMC was consistently activated across both task types, regardless of motor execution confounds. This result thus reveals a key role for the left dPMC in sensorimotor integration and response selection during motor learning tasks. The results of the present meta-analysis thus indicate that the left dPMC is a key structure in the network of brain areas that underlie motor learning.

## Figures and Tables

**Table 1:** Coordinates of peak activations revealed in the global analysis.

Macroanatomical location	Cytoarchitectonic location	Cluster vol. (mm^3^)	Z-score	MNI coordinates		
x	y	z
*Global analysis: all tasks (n = 70)*						
L dPMC	Area 6	1050	5.65	− 32	− 12	60
L M1	Area 4a		5.52	− 38	− 24	58
L SMC	Area 6	870	5.89	0	− 2	56
R SMC	Area 6		5.41	2	8	52
R cerebellum	Lobules V–VI	467	4.13	10	− 58	− 20
R cerebellar vermis	Lobules I–IV		3.69	0	− 54	− 12
L thalamus		350	5.76	− 12	− 20	10
R M1	Area 4a	348	4.08	40	− 20	54
R S1	Area 3b		3.55	32	− 24	62
L putamen		302	5.20	− 26	4	4
L SPL	Area 7a	243	4.46	− 30	− 56	64
R putamen		194	4.54	26	0	2
L cerebellum	Lobule VI	134	4.60	− 20	− 52	− 22

*Subanalysis: all right handed experiments (n = 39)*						
L SMC	Area 6	448	5.52	− 2	12	54
L M1	Area 4a		5.07	− 38	− 24	56
L dPMC	Area 6	366	6.48	− 26	4	62
L dPMC	Area 6	341	4.63	− 36	− 14	60
L Thal	Th-Prefrontal	315	5.60	− 12	− 20	10
L SPL	Area 7A	209	4.44	− 30	− 56	64
R dPMC	Area 6	157	4.87	38	6	62
R S1	Area 2	142	4.13	34	− 40	54
R SPL	SPL 7PC		3.89	44	− 48	60
L putamen		114	4.13	− 26	4	2

*Subanalysis: all movement controlled experiments (n = 47)*						
L dPMC	Area 6	353	6.01	− 26	4	62
R dPMC	Area 6	185	2.79	24	− 4	62
L thalamus		181	5.22	− 12	− 20	10
L SPL	Area 7A	175	4.18	− 26	− 58	58
R SMC	Area 6	144	4.10	2	4	54

**Table 2:** Coordinates of peak activations revealed in the task specific analysis of sensorimotor tasks.

Macroanatomical location	Cytoarchitectonic location	Cluster vol. (mm^3^)	Z-score	MNI coordinates		
x	y	z
*Sensorimotor tasks (n = 35)*						
L dPMC	Area 6	518	4.10	− 30	− 14	62
L M1			4.94	− 38	− 24	62
RM1		289	3.91	34	− 22	62
R S1	Area 3b		3.95	40	− 20	50
L SMC	Area 6	229	4.72	− 2	− 4	56
R SMC	Area 6	229	3.42	2	− 6	68
R cerebellar vermis	Lobules I–IV	261	4.12	2	− 54	− 12
R putamen		224	4.49	26	10	4
L putamen		191	4.64	− 26	2	0
R cerebellum	Lobules V–VI	176	5.67	28	− 50	− 30
L cerebellum	Lobule VI	156	4.65	− 20	− 52	− 24

*Subanalysis: right handed sensorimotor task experiments (n = 20)*						
L dPMC	Area 6	313	3.82	− 34	− 12	60
L M1	Area 4a		3.96	− 40	− 20	54
R putamen		157	4.25	− 26	2	2
L putamen		96	4.88	26	10	4

*Subanalysis: movement controlled sensorimotor task experiments (n = 23)*						
L dPMC	Area 6	116	3.94	− 26	4	58

**Table 3:** Coordinates of peak activations revealed in the task specific analysis of SRTT variants.

Macroanatomical location	Cytoarchitectonic location	Cluster vol. (mm^3^)	Z-score	MNI coordinates		
x	y	z
*SRTT variants (n = 35)*						
R SMC	Area 6	612	5.27	2	8	52
L SMC	Area 6		3.73	2	20	44
L dPMC	Area 6	192	5.34	− 26	6	64
L thalamus		178	5.61	− 12	− 20	10
L M1		162	4.49	− 34	− 12	60
R cerebellum	Lobule VI	153	5.55	24	− 54	− 20
L SPL	Area 7A	138	3.95	− 30	− 56	64
R dPMC	Area 6	97	4.59	38	6	62

*Subanalysis: right handed SRTT variants (n = 19)*						
L SMC	Area 6	245	5.25	0	12	52
L dPMC	Area 6	189	6.00	− 26	6	64
L thalamus		156	5.01	− 10	− 18	12
R dPMC		148	5.34	38	6	62
L M1	Area 4	134	3.98	− 38	− 24	56
L S1	Area 1		3.84	− 40	− 32	64
R visual cortex	V3V	113	4.28	14	− 92	− 14
R vPMC	Area 44/45	105	4.75	42	12	30

*Subanalysis: movement controlled SRTT variants (n = 24)*						
L dPMC	Area 6	183	5.85	− 26	6	64
L thalamus	Th-Prefrontal	149	5.13	− 12	− 20	10
R dPMC	Area 6	131	5.14	38	6	62
R cerebellum	Lobule VI	115	5.00	24	− 54	− 20

**Table 4:** Coordinates of peak activations revealed in the conjunction analyses.

Macroanatomical location	Cytoarchitectonic location	Cluster vol. (mm^3^)	Z-score	MNI coordinates		
x	y	z
*Conjunction: all tasks, sensorimotor tasks, SRTT variants*						
L SMC	Area 6	101	4.10	0	− 2	54
L dPMC	Area 6	88	3.90	− 32	− 12	60
L M1		50	3.90	− 26	2	60
R cerebellum	Lobule VI	16	3.38	26	− 54	− 26

*Conjunction: right handed subanalyses*						
L M1	Area 4a	47	3.27	− 38	− 24	56
L dPMC	Area 6	41	3.78	− 26	2	60

*Conjunction: movement controlled subanalyses*						
L dPMC	Area 6A	42	3.69	− 26	4	60

*Combined conjunction analysis*						
L dPMC	Area 6A	35	3.69	− 26	4	60
