# Supplementary material for: A quantitative meta-analysis and review of motor learning in the human brain
Source: Neuroimage. 2013 Feb 15;67:283–97. doi: 10.1016/j.neuroimage.2012.11.020 (PMC3555187; doi:10.1016/j.neuroimage.2012.11.020)
Supplement: Supplementary file 1 — Supplementary material [file mmc1.doc]

| **Supplementary Table 1:** Experiments included in the meta-analysis | | | | | | |
| --- | --- | --- | --- | --- | --- | --- |
| ***Sensorimotor tasks*** | | | | | | |
| **First Author, Year** | **Mode** | **Sub** | **Paradigm** | **Hand** | **Reported Contrast** | **Foci** |
| Anguera, 2007 | fMRI | 11 | Adapting joystick movements to 30° rotated visual feedback | Right | Early adaptation | 10 |
|  | fMRI | 11 | Adapting joystick movements to 30° rotated visual feedback | Right | Conjunction of Early & late adaptation | 4 |
| Buccino, 2004 | fMRI | 12 | Guitar chord imitation | Left | Task>rest | 13 |
| Debaere, 2004 | fMRI | 12 | Perform 90ϕ wrist movements before & after training | Bimanual | Post>pre | 15 |
| Debas, 2010 | fMRI | 48 | Adapting joystick movements to 180°rotated visual feedback | Right | BOLD increase post training | 17 |
| Dieber, 1997 | PET | 6 | conditional motor task with nonspatial and spatial rules | Right | Task>control | 8 |
|  | PET | 6 | respond to spatial rule/conditional motor task | Right | Task>control | 2 |
| Duff, 2007 | fMRI | 12 | Finger thumb opposition | Left | Increase in OSORU signal | 19 |
| Fischer, 2005 | fMRI | 8 | Dexterous finger-thumb opposition task | Left | Task>rest | 21 |
| Floyer-Lea, 2004 | fMRI | 15 | Force transducer controlled visual tracking task | Right | Task>rest | 20 |
| Grafton, 2001 | PET | 7 | Pursuit tracking task | Right | Increase in rCBF | 7 |
| Inoue, 1997 | PET | 6 | Adapting reaching movements to 60° rotated visual feedback | Right | Early adaptation>normal reaching | 4 |
|  | PET | 6 | Adapting reaching movements to 60° rotated visual feedback | Right | Late adaptation>normal reaching | 5 |
| Inoue, 2000 | PET | 6 | Adapting reaching movements to 60° rotated visual feedback | Right | Early adaptation>normal reaching | 9 |
|  | PET | 6 | Adapting reaching movements to 60° rotated visual feedback | Right | Late adaptation>normal reaching | 10 |
| Jantzen, 2002 | fMRI | 8 | Squeezing a ball in synchronization with a metronome | Right | Trained>rest | 5 |
|  | fMRI | 8 | Squeezing a ball in syncopation with a metronome | Right | Trained>rest | 7 |
| Kawashima., 1998 | PET | 8 | Two ball rotation task | Bimanual | Trained>rest | 18 |
| Krakauer, 2004 | PET | 12 | Adapting hand movements to 30° rotated visual feedback | Right | Task>control | 3 |
| Matsumura, 2004 | PET | 13 | Two ball rotation task | Right | Increase rCBF | 6 |
|  | PET | 13 | Two ball rotation task | Left | Increase rCBF | 7 |
| Miall, 2001 | fMRI | 9 | Cursor tracking with joystick | Right | Task>rest | 8 |
| Nakamura, 2001 | PET | 8 | Joystick controlled cursor movements to sequence of targets | Right | Task>control | 4 |
| Puttemans, 2005 | fMRI | 11 | Wrist movements at a 2:1 frequency | Bimanual | BOLD increases across sessions | 4 |
| Remy, 2008 | fMRI | 12 | 90ϕ wrist movements before & after training | Bimanual | Post>pre | 2 |
|  | fMRI | 12 | Interactions between 0ϕ & 90ϕ wrist movements | Bimanual | Post>pre | 2 |
| Remy, 2010 | fMRI | 12 | 90ϕ wrist movements after training | Bimanual | Post>rest | 20 |
|  | fMRI | 12 | 90ϕ wrist movements with distraction task after training | Bimanual | Dual task post>rest | 24 |
| Ronsse, 2010 | fMRI | 38 | 90ϕ wrist movements after training, collapsed across groups | Bimanual | Post>pre | 1 |
|  | fMRI | 38 | 90ϕ wrist movements after training + augmented feedback | Bimanual | Post>pre | 8 |
| Seidler, 2006 | fMRI | 26 | Adapting joystick movements to 30°/45° visual rotation | Right | Early adaptation>control | 12 |
|  | fMRI | 26 | Adapting joystick movements to 30°/45° visual rotation | Right | Adaptation>control | 5 |
| Tracy, 2001 | fMRI | 5 | Finger thumb opposition | Right | Post>pre | 19 |
| Tracy, 2003 | fMRI | 15 | Tying knots (strong improvement in performance) | Bimanual | Post>pre | 2 |
|  | fMRI | 15 | Tying knots (weak improvement in performance) | Bimanual | Post>pre | 2 |
| ***SRTT Variants*** | | | | | | |
| **First Author, Year** | **Mode** | **Sub** | **Paradigm** | **Hand** | **Reported Contrast** | **Foci** |
| Bischoff-Grethe, 2004 | fMRI | 16 | 12 item explicit SRTT with variable response mapping. | Right | Task>baseline | 39 |
| Cross, 2007 | fMRI | 27 | 4 item explicit SRTT go/no go task | Left | Task>baseline | 34 |
| Daselaar, 2003 | fMRI | 26 | 12 item implicit SRTT in younger (mean age 32.4) adults. | Bimanual | Sequence>random | 18 |
|  | fMRI | 34 | 12 item implicit SRTT in older (mean age 66.4) adults. | Bimanual | Sequence>random | 24 |
| Debas, 2010 | fMRI | 48 | 5 item explicit SRTT | Left | BOLD increase post training | 12 |
| Fernandez-Seara, 2009 | fMRI | 14 | 6 item explicit SRTT | Right | Early learning>rest | 44 |
|  | fMRI | 14 | 6 item explicit SRTT | Right | Learning related rCBF changes | 33 |
| Garraux, 2005 | fMRI | 15 | 5 item explicit SRTT based on memorized sequences | Right | Task>rest | 30 |
| Gheysen, 2010 | fMRI | 22 | 5 item implicit SRTT using a color matching task | Bimanual | Linear interaction U Sequence>random | 1 |
| Grafton, 1995 | PET | 12 | 6 item implicit SRTT with auditory distraction task | Right | Increase in rCBF | 7 |
|  | PET | 12 | 6 item implicit SRTT without auditory distraction task | Right | Increase in rCBF | 8 |
| Grafton, 2002 | PET | 8 | 6 item implicit SRTT with auditory distraction task | Left hand | Increase in rCBF | 22 |
| Haaland, 2004 | fMRI | 14 | 5 item explicit SRTT (contrast collapsed across hands) | Bimanual | Complex>simple | 9 |
| Heun, 2004 | fMRI | 10 | 15 item implicit SRTT | Right | Sequence>random | 19 |
| Landau, 2006 | fMRI | 8 | Modified SRTT | Bimanual | Sequence>random | 7 |
| Muller, 2002 | fMRI | 7 | 6 item explicit SRTT | Right | Novel complex sequence > index finger | 20 |
|  | fMRI | 7 | 6 item explicit SRTT | Right | Novel complex sequence > simple sequence | 31 |
|  | fMRI | 7 | 8 item explicit SRTT | Right | Early sequence learning > simple sequence | 21 |
|  | fMRI | 7 | 8 item explicit SRTT | Right | Late sequence learning > simple sequence | 23 |
| Muller, 2003 | fMRI | 8 | 6 item explicit SRTT | 7 Right, 1 Left | Novel complex sequence > index finger | 17 |
|  | fMRI | 8 | 6 item explicit SRTT | 7 Right, 1 Left | Novel complex sequence > simple sequence | 25 |
| Nasmith, 2010 | fMRI | 20 | 12 item implicit SRTT | Bimanual | Sequence>random | 21 |
| Olson, 2006 | fMRI | 10 | 11 item implicit SRTT | Bimanual | Task>rest | 14 |
| Orban, 2010 | fMRI | 16 | 8 item explicit SRTT | Left | Fixed>random | 6 |
| Parsons, 2005 | fMRI | 12 | 4 item explicit SRTT with varying response associations | Right | Task>rest | 4 |
| Poldrack, 2005 | fMRI | 14 | 12 item implicit SRTT & auditory distraction task, pre training | Right | Task>rest | 7 |
|  | fMRI | 14 | 12 item implicit SRTT& auditory distraction task, post training | Right | Task>rest | 6 |
| Schubotz, 2001 | fMRI | 12 | 3 item explicit SRTT variation | Bimanual | Task>control | 20 |
| Seidler, 2002 | fMRI | 6 | 12 item implicit SRTT with secondary (distracting) task | Right | Fixed>random | 9 |
| Steele, 2010 | fMRI | 15 | Temporal motor sequencing task | Right | Day 2 > day 1 | 36 |
|  | fMRI | 15 | Temporal motor sequencing task | Right | Day 5 > day 2 | 14 |
| Van der Graaf, 2004 | fMRI | 12 | 6 item implicit double SRTT | Bimanual | Increases in BOLD within session | 9 |
|  | fMRI | 6 | 6 item implicit double SRTT | bimanual | Increases in BOLD between sessions | 20 |
| Weirheid, 2003 | fMRI | 7 | 12 item implicit SRTT | Bimanual | Fixed>random | 7 |
| Willingham, 2002 | fMRI | 18 | 12 item explicit SRTT | Bimanual | Fixed>random | 14 |

NOTE: ‘rest’ describes conditions in which participants do not perform movements, and includes conditions with passive tactile or visual stimulation.

**Supplementary Table 2: Sub Analysis of Right handed tasks**

| **Macroanatomical**  **location** | **Cytoarchitectonic**  **location** | **Cluster Vol. (mm^3^)** | **Z-score** | **MNI coordinates** | | |
| --- | --- | --- | --- | --- | --- | --- |
|  |  |  |  | **x** | **y** | **z** |
| *All Right Handed Tasks* | | | | | | |
| L SMC | Area 6 | 448 | 5.52 | -2 | 12 | 54 |
| L dPMC | Area 6 | 366 | 6.48 | -26 | 4 | 62 |
| L M1 | Area 4a | 341 | 5.07 | -38 | -24 | 56 |
| L Thal | Th-Prefrontal | 315 | 5.60 | -12 | -20 | 10 |
| L SPL | Area 7A | 209 | 4.44 | -30 | -56 | 64 |
| R dPMC | Area 6 | 157 | 4.87 | 38 | 6 | 62 |
| R S1 | Area 2 | 142 | 4.13 | 34 | -40 | 54 |
| R SPL | SPL 7PC |  | 3.89 | 44 | -48 | 60 |
| L Putamen |  | 114 | 4.13 | -26 | 4 | 2 |
|  |  |  |  |  |  |  |
| *Right Handed SRTT Variants* | | | | | | |
| L SMC | Area 6 | 245 | 5.25 | 0 | 12 | 52 |
| L dPMC | Area 6 | 189 | 6.00 | -26 | 6 | 64 |
| L Thalamus |  | 156 | 5.01 | -10 | -18 | 12 |
| R dPMC |  | 148 | 5.34 | 38 | 6 | 62 |
| L M1 | Area 4 | 134 | 3.98 | -38 | -24 | 56 |
| L S1 | Area 1 |  | 3.84 | -40 | -32 | 64 |
| R Visual Cortex | V3V | 113 | 3.64 | 22 | -82 | -12 |
| R vPMC | Area 44/45 | 105 | 4.57 | 42 | 12 | 30 |
|  |  |  |  |  |  |  |
| *Right Handed Sensorimotor tasks* | | | | | | |
| L dPMC | Area 6 | 313 | 4.77 | -26 | 0 | 58 |
| L M1 | Area 4a |  | 4.25 | -40 | -20 | 54 |
| L Putamen |  | 157 | 5.24 | 26 | 10 | 4 |
| R Putamen |  | 96 | 4.42 | -26 | 2 | 2 |
|  |  |  |  |  |  |  |
| *Right Handed Conjunction Analysis* | | | | | | |
| L dPMC | Area 6 | 47 | 3.78 | -26 | 2 | 60 |
| L M1 | Area 4a | 41 | 3.27 | -38 | -24 | 56 |

**Supplementary Table 3: Sub Analysis of experiments that controlled for movement execution**

| **Macroanatomical**  **location** | **Cytoarchitectonic**  **location** | **Cluster Vol. (mm^3^)** | **Z-score** | **MNI coordinates** | | |
| --- | --- | --- | --- | --- | --- | --- |
|  |  |  |  | **x** | **y** | **z** |
| *All movement controlled tasks* | | | | | | |
| L dPMC | Area 6 | 353 | 6.01 | -26 | 4 | 62 |
| R dPMC | Area 6 | 185 | 2.79 | 24 | -4 | 62 |
| L Thalamus |  | 181 | 5.22 | -12 | -20 | 10 |
| L SPL | Area 7A | 175 | 4.18 | -26 | -58 | 58 |
| R SMC | Area 6 | 144 | 4.10 | 2 | 4 | 54 |
|  |  |  |  |  |  |  |
| *Movement controlled SRTT Variants* | | | | | | |
| L dPMC | Area 6 | 183 | 5.85 | -26 | 6 | 64 |
| L Thalamus | Th-Prefrontal | 149 | 5.13 | -12 | -20 | 10 |
| R dPMC | Area 6 | 131 | 5.14 | 38 | 6 | 62 |
| R Cerebellum | Lobule VI | 115 | 5.00 | 24 | -54 | -20 |
|  |  |  |  |  |  |  |
| *Movement controlled Sensorimotor tasks* | | | | | | |
| L dPMC | Area 6 | 116 | 3.94 | -26 | 4 | 58 |
|  |  |  |  |  |  |  |
| Movement controlled Conjunction analysis | | | | | | |
| L dPMC | Area 6 | 35 | 3.69 | -26 | 4 | 60 |

**Figure S1**: Analysis of right handed experiments that also controlled for movement execution


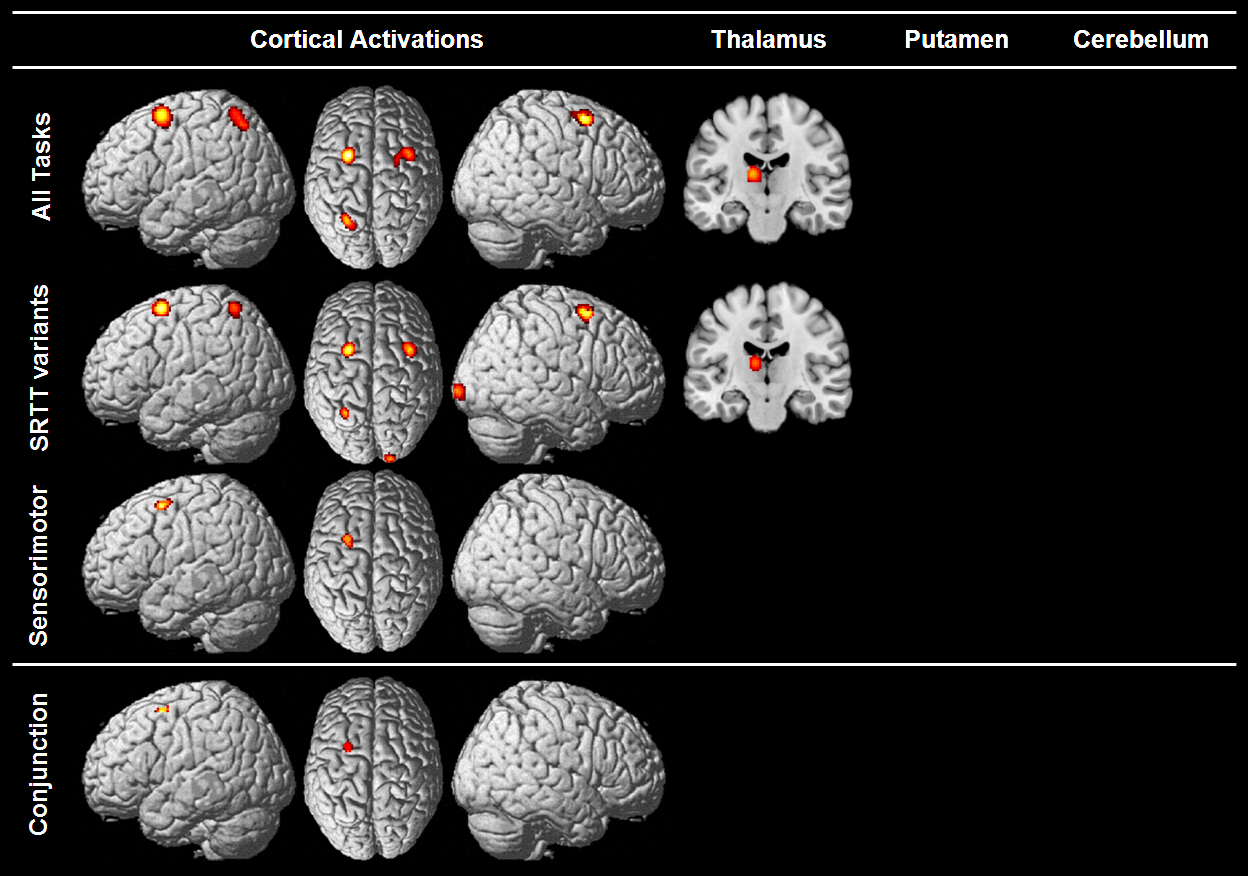


The analysis of right handed tasks that controlled for movement execution had only a total of 12 studies, and is thus likely to be underpowered. It is, however, of interest to note the converging left dPMC activity, which is consistent with the data presented in the main manuscript. Only the thalamus was found to be activated at the subcortical level, consistent with its proposed role as a relay station.

**Training related decreases in activity**

**
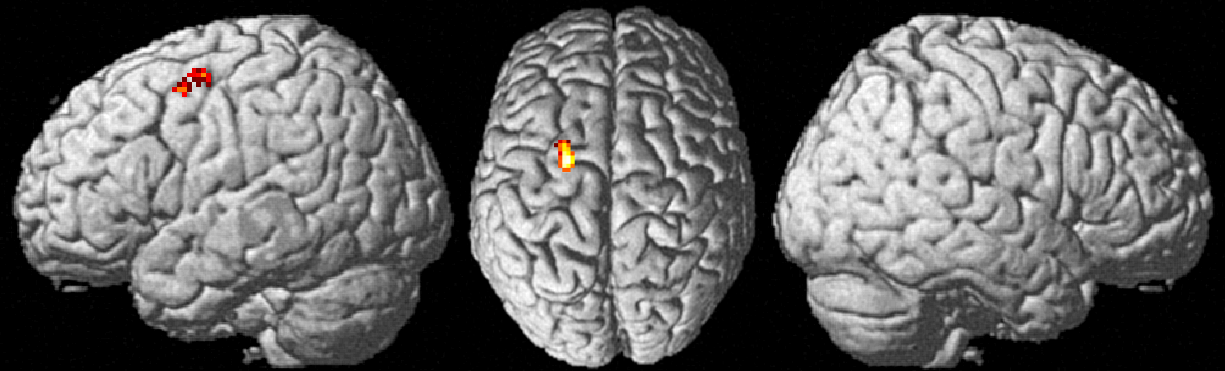
**

**Figure S2:** Training related decreases in activity **t**hresholded at the P<0.05, cluster-level FWE, corrected for multiple comparisons, cluster-forming threshold at voxel level p<0.001.


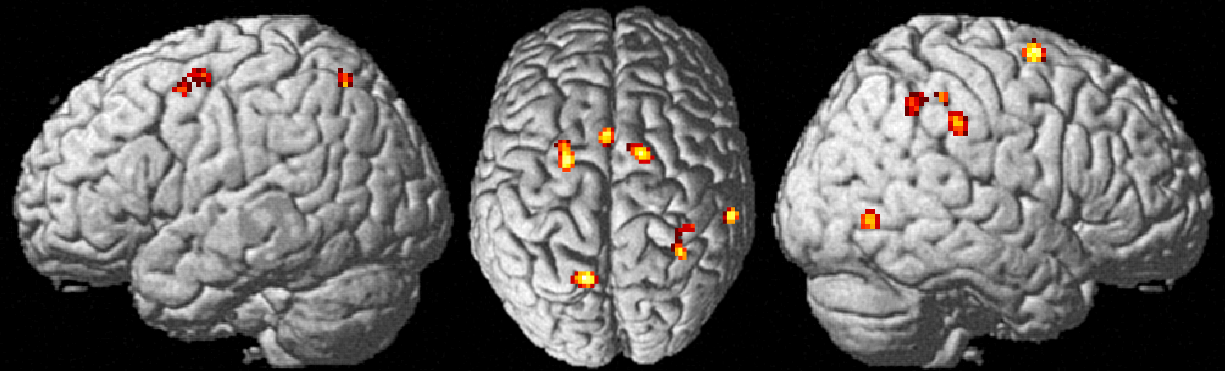


**Figure S3:** As Figure S2, but at the cluster level uncorrected P<0.05 significance criterion.

An analysis of training related decreases in activity revealed only a single cluster of activity in the left superior/middle frontal gyrus when correcting for multiple comparisons across the whole brain (see Figure S2). Given the rather low number of experiments and hence low power of this analysis, Figure S3 presents an exploratory inference based on a more lenient significance criterion (cluster-level uncorrected p<0.05, while keeping the cluster-forming threshold at p<0.001 at voxel level). This revealed additional activations in the left SMA, left cerebellar vermis (lobule V), right middle temporal gyrus, left superior parietal lobule, right inferior parietal lobule, and the inferior frontal gyrus.

It is of note that some areas identified in this analysis were also implicated in training related increases in activity (i.e. the SMA and cerebellum). We attribute this result to the differing time-courses of their activation during motor learning. For instance, Nezfat et al., (2001) found that the cerebellum was activated strongly during early phases of sensorimotor learning (when error was high), but that its activation decreased during later phases of learning (when error was low).

As our meta-analysis is limited to the contrasts reported in previous studies, we are unlikely to be able to detect such subtle fluctuations in brain activity over time. This could therefore explain why the SMA and cerebellum showed both training related increases in activity, and (less statistically reliable) training related decreases in activation.
